# The role of animal-assisted programs in physical health improvement of children and adolescents with special education needs - a systematic review

**DOI:** 10.1186/s12889-024-18326-y

**Published:** 2024-03-15

**Authors:** Karolina Eszter Kovács, Éva Zita Balogh, Buda Lovas, Péter Boris, Beáta Erika Nagy

**Affiliations:** 1 Faculty of Arts, Institute of Psychology, University of Debrecen, Debrecen, Hungary; 2 Doctoral School of Human Sciences, Doctoral Program on Psychology, University of Debrecen, Debrecen, Hungary; 3 Eötvös Loránd University, Budapest, Hungary; 4 Laki Kálmán Doctoral School, University of Debrecen, Debrecen, Hungary; 5 Faculty of Medicine, Institute of Pediatrics, Pediatric Rehabilitation, Pediatric Psychology and Psychosomatic Unit, head of the Unit, University of Debrecen, Debrecen, Hungary

**Keywords:** Animal-assisted programs, Physical health, Children with special needs, Systematic review

## Abstract

Programs involving animals in therapeutic programs are becoming increasingly prevalent. These programs can vary greatly in their approach, scope, and objectives, and they can significantly impact the development of healthy children and those with various disorders. In this systematic review, we sought to investigate the psychological ramifications of animal-assisted activities (AAA), therapies (AAT), and interventions (AAI). We searched for relevant studies using the EBSCO Discovery Service search engine across 85 databases, utilising appropriate keywords. Our search generated 262 results, of which 21 were selected for inclusion after title and abstract screening, as well as full-text analysis. Our findings indicate that dogs and horses are animal-assisted programs’ most commonly used animals. Additionally, autism, cerebral palsy, and ADHD were found to be overrepresented in these programs. Furthermore, the length of sessions and overall program duration exhibited considerable variation, regardless of patient age or disease type. The principal measures centred on the physiological variables related to the nervous system and motorium-related indicators. The studies were generally of exceptional methodological soundness. Frequently, the studies narrowed their scope to a single segment or just the child or adolescent, but the outcomes lacked contextual interpretation. Expanding the range of studies by comparing psychological and physiological indicators and conducting follow-up analysis with a longitudinal design would be beneficial.

**Supplementary Information:**

The online version contains supplementary material available at 10.1186/s12889-024-18326-y.

## Background

Animal-assisted programs have significant relevance in various settings due to their diverse positive impact on individuals and communities. Animals provide a non-judgmental and non-threatening presence, which can help individuals, especially those with social difficulties or autism spectrum disorders, practice their communication skills while they do not receive any negative feedback despite making mistakes [1]. Also, animals communicate primarily through non-verbal cues, such as body language and facial expressions which can be comforting for individuals who struggle with verbal communication [2]. In addition, participating in activities with animals often leads to increased social interaction both with the animal and with people who share similar interests [3]. Animal-assisted activities and therapies can be adapted to a wide range of settings, including hospitals [4], nursing homes [5], schools [6], prisons [7], and rehabilitation centres [8]. Interacting with animals often requires problem-solving skills, memory recall, and learning, which can stimulate cognitive functions [3, 9]. In some cases, animal-assisted activities can provide an alternative or complementary approach to traditional medical or psychological interventions [10, 11].

Animal-assisted activities, therapies and interventions have become popular due to their various positive impact. Emotional and psychological benefits are regularly reported during such programs: interacting with animals has been shown to reduce levels of stress and anxiety and can improve mood and happiness from the psychological aspect of animal-assisted therapies [12, 13]. Regarding physical benefits, engaging with animals, such as walking dogs or grooming horses, can lead to increased physical activity. This can positively affect effect cardiovascular health, weight management, and overall physical fitness [14, 15]. Some studies have highlighted that interactions with animals can decrease the perception of pain, particularly in clinical settings, increase muscle strength and improve control of fine motor skills [16, 17].

This paper aims to systematically explore previous studies that have assessed the impact of animal-assisted activities, interventions and therapies on physical health characteristics, focusing on children and adolescents with special education needs. In our research, we to examine areas of research involving animal-assisted programs to improve physical-health development in terms of implementation, practical implications, and areas in need of additional research. These programs offer numerous advantages and benefits. However, there are no guidelines or best practices for how these practices should be carried out. A review may be a useful resource to synthesize information for practitioners and detail what has been done and what is in need of further research for researchers. Towards that the end, we conducted a systematic review to examine areas of research involving animal-assisted programs to improve physical-health development in terms of implementation, practical implications, and areas in need of additional research. Specifically, we sought to understand (a) common health outcomes addressed by animal-assisted interventions, (b) how animal-assisted interventions are carried out, (c) specific implications for practice, and (d) areas in need of further research.

## Methods

This systematic literature review follows the Preferred Reporting Items for Systematic Reviews and Meta-Analyses (PRISMA) guidelines [18]. The current systematic review is registered in the International Platform of Registered Systematic Review and Meta-analysis Protocols (INPLASY, https://inplasy.com/inplasy-2024-1-0090/, 10.37766/inplasy2024.1.0090).

**Fig. 1:**
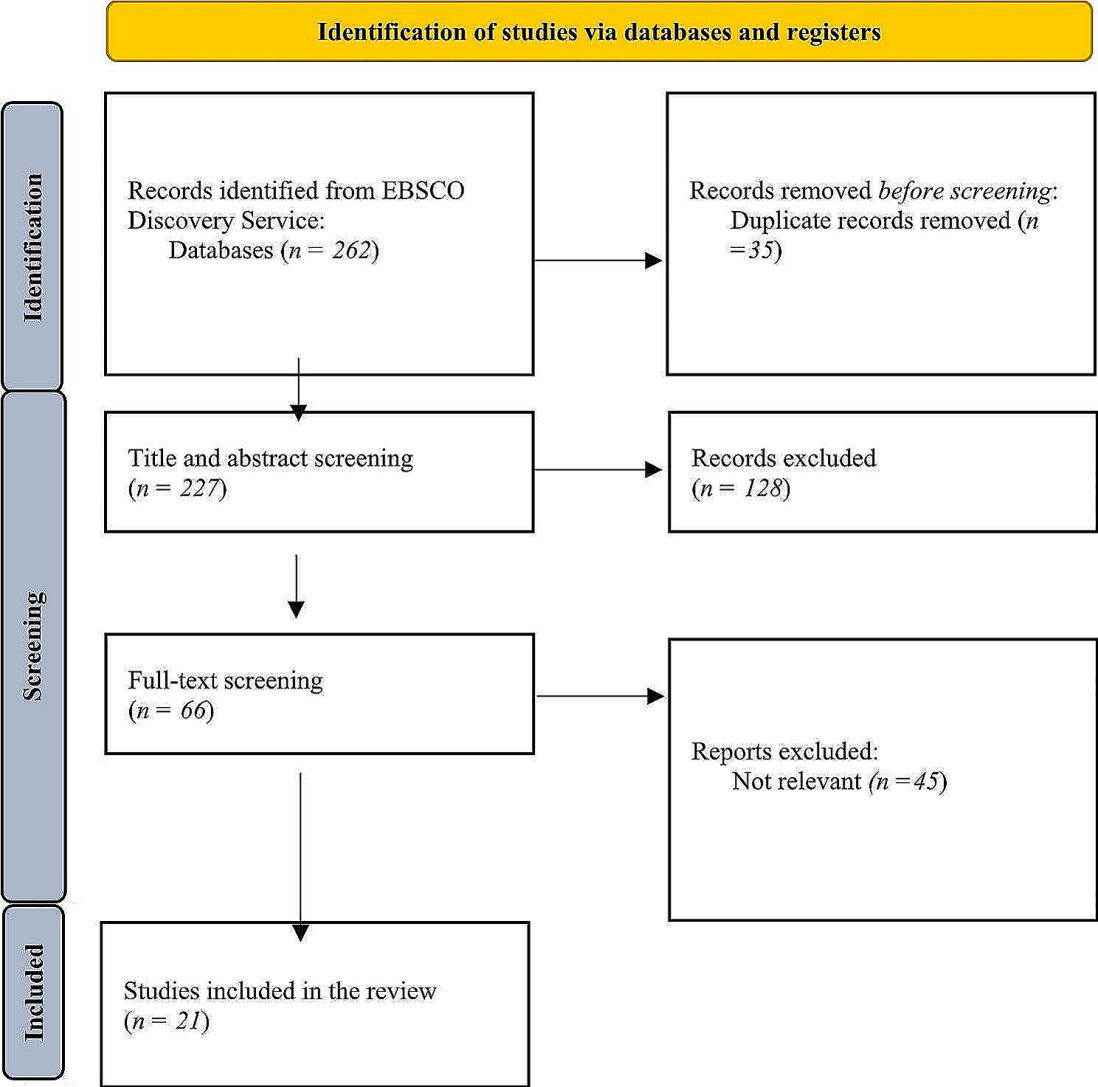
Preferred reporting items for systematic reviews and meta-analyses (PRISMA) diagram

### Literature search

We used the EBSCO (Elton B. Stephens CO (company)) Discovery Service Search Engine for systematic search, which contains 85 databases. The keywords we used for searching were “animal-assisted therapy”, “animal-assisted activity” OR “animal-assisted intervention” OR “pet therapy” AND “children” AND “special education” AND “psychological intervention”. These terms were searched by using the “All text” option during the systematic search. The systematic searches, which has been carried out between 12 and 19 July 2023, found 262 records (all records were searched). After double filtering, we excluded 35 records, and a further 128 records were excluded after title and abstract screening, overall, 66 papers were included in full-text screening, and 21 papers were involved in the qualitative synthesis.

### Inclusion and exclusion criteria

During the screening, we set a list of inclusion criteria. To be included, studies must be original empirical research published in English in peer-reviewed journals. We considered only empirical research, including both exploratory studies (e.g., pilot, experience) and comparative studies (controlled and non-controlled trials, between-group comparisons). Study participants were below 18 years of age with special needs, diagnosed according to DSM or BNO criteria. Studies focusing on pet ownership or casual animal interactions were excluded. We excluded reviews, commentaries, letters to the editor, conference papers, books, book chapters, dissertations, and newspaper articles. Additionally, papers involving only children without special needs were not considered.

### Data extraction and assessment of methodological quality

We performed a multistage screening process to select studies which met the inclusion criteria. In the first step, the first author (KEK) searched the literature. In the next stage, the first review author screened the titles and abstracts of all identified records (KEK), and twenty-five per cent of all titles and abstracts were independently assessed by a second review author (EZB, BEN, BL, PB). Therefore, all titles and abstracts were checked by two authors. Besides the papers that unquestionably passed this screening stage, all studies whose appropriateness in the research context was questionable were taken forward to the full-text screening at this stage. In the next step, full-text screening was performed, in which the authors (KKE, EZB, BEN, BL) independently screened all full texts. In cases of uncertainty (when it was not evident to send the article for analysis due to the focus or the paper or its special sample, e.g. children after surgery), the other authors also checked the decision.

For data extraction, an Excel spreadsheet and Data Extraction Forms were applied. Variables investigated during the analysis and their definitions are introduced in Table 1. We included full article citation, study objectives, study design, how the study attempted to avoid bias, participant characteristics and numbers, intervention and/or comparison, results/outcome and comments related to study quality.

**Table 1:** Variables investigated during the analysis and their definitions

Variable	Definition
Sample	participants below 18 years of age with special needs, diagnosed according to DSM or BNO criteria, see ‘disorders’, number of participants and their age was noted (see *3.2. Age of the patients and the special needs represented)*
Disorder	the type of disorder the child owned, i.e. attention deficit or/and hyperactivity disorder, autism cerebral palsy, developmental delay, developmental dysphasia dyspraxia, neurological disorder, physiological disorders, physical disability
Study design	design used to answer a particular research question is determined by the nature of question, i.e. randomized-controlled trial, non-randomized controlled trial, cluster randomized trial, pilot (not specified), survey
Animal	the animal involved in the program, i.e. dog, horse (see *3.1. Animals involved in the therapies)*
Session duration	the length of the sessions given in minutes (see *3.3. Session and Program Duration by Age)*
Program duration	the length of the program given in weeks or months (see *3.3. Session and Program Duration by Age)*
Outcome variables	research outputs (physiological indicators) measured in the programs were collected to conduct content analysis regarding the focus of the research to determine and categorise the main focus of development (see *3.4. Outcome indicators*); categorisation was carried out parallelly and independently by three authors (KEK, ÉZB, BL), then the authors checked the category system and the categories, and their content were unified at the end of the process

### Risk of bias

As the critical appraisal tool to check the risk of bias, the Joanna Briggs Institute (JBI) critical appraisal tool was applied (randomised controlled trials and non-randomised controlled trials followed by Barker et al. [4] and cross-sectional studies followed by Moola et al. [19]). This tool is developed by the JBI Effectiveness Methodology Group to support the process of critical appraisal which must be carried out during systematic literature reviews. Papers were evaluated using the appropriate tool on a 4-point Likert scale (yes/no/unclear/not applicable) (Appendix, Supplementary Table 1 and Supplementary Table 2).

**Table 2:** Papers involved in the systematic review and their most important characteristics

	Disorder	Type	Animal	Session length	Program length	Sample (N)	Age	Main outcome	Additional outcomes
Abadi et al. 2022 [20]	autism	RCT	dog	60 min	7 weeks	20	6–14 years	physical activity	N/A
Benda et al. 2003 [21]	cerebral palsy	RCT	horse	8 min	N/A	15	4–12 years	brain activity	N/A
Brady et al. 2021 [22]	developmental delay	N/A	horse	15–30 min	N/A	N/A	2–3 years	gross motor development	N/A
Branson et al. 2017 [23]	physiological disorders	RCT	horse	10 min	10 months	48	7–17 years	salivary cortisol, CRP	anxiety, mood
Cabiddu et al. 2016 [24]	neurological disorder	trial	horse	50 min	N/A	12	4–12 years	anthropometric measurements, heart rate, respiratory rate, peripheral oxygen saturation (SpO2), systolic and diastolic blood pressure	N/A
Calcaterra et al. 2015 [25]	unspecified, post-surgery	RCT	dog	20 min	1 occasion	40	3–17 years	physical stress	anxiety
Deutz et al. 2018 [26]	cerebral palsy	RCT	dog	16–20 min	N/A	73	5–15 years	gross motor development	quality of life
Hession et al. 2014 [27]	dyspraxia	pilot	horse	30 min	8 weeks	40	6–15 years	ambulation	cognition, mood
Hsieh et al. 2017 [28]	cerebral palsy	pilot	horse	30 min	12 weeks	14	3–8 years	body functions, activities and participation	N/A
Jang et al. 2018 [29]	attention deficit or/and hyperactivity disorder	pilot	horse	30 min	12 weeks	20	7–11 years	motor proficiency	behaviour, self-esteem
Kraft et al. 2019 [30]	neurological disorder	RCT	horse	45–60 min	12 weeks	5	2–5 years	neurodevelopmental progression	N/A
Kwon et al. 2015 [31]	cerebral palsy	RCT	horse	30 min	8 weeks	91	4–10 years	gross motor development	N/A
Lucena-Antón et al. 2018 [32]	cerebral palsy	RCT	horse	45 min	12 weeks	44	3–14 years	muscle spasticity	N/A
Machová et al. 2019 [33]	developmental dysphasia	RCT	dog	46 min	10 months	69	4–6 years	facial motricity, motor proficiency	N/A
Oh et al. 2018 [34]	attention deficit or/and hyperactivity disorder	RCT	horse	60 min	12 weeks	32	6–12 years	coordination, brain activity	self-esteem, quality of life
Park et al. 2021 [35]	cerebral palsy	RCT	horse	40 min	16 weeks	26	6–12 years	resting heart rate (RHR), peak oxygen uptake (VO2peak)	N/A
Rincón et al. 2021 [36]	severe and multiple disabilities	RCT	dog	45 min	12 weeks	14	3–12 years	postural, oculomotor, language and autonomy	N/A
Silkwood-Sherer & McGibbon 2022 [37]	cerebral palsy	RCT	horse	45 min	12 weeks	13	3–6 years	motor function	quality of life
Silkwood-Sherer et al. 2012 [38]	disabled	RCT	horse	45 min	6 weeks	16	5–16 years	motor function	N/A
Steiner & Kertesz 2015 [39]	autism	RCT	horse	30 min	4 weeks	26	10–13 years	gait analysis, skills	N/A
Žalienė et al. 2018 [40]	cerebral palsy	pilot	horse	N/A	2 weeks − 4 years	15	3–19 years	gross motor function	N/A

*Note* N/A: not accessible, RCT: randomized controlled trial, CRP = c-reactive protein

## Results

Figure 1 shows the process of the systematic analysis. A total of 21 articles met the criteria. The articles were published between 2003 and 2022. Most studies (*N* = 7) were investigated in the United States [21–23, 30, 31, 37, 38]. Three programs were investigated in Korea [29, 34, 35] and two in Spain [32, 36]. Also, the following countries were investigated by one study: Brazil [24], Canada [20], Czech Republic [33], Germany [26], Hungary [39], Ireland [27], Italy [25], Lithuania [40], and Taiwan [28]. Therefore, nine programs were carried out in America, 8 in Europe and 4 in Asia (see Table 2).

Regarding the topic, three main groups of dependent variables could be seen created by content analysis. Firstly, most research focused on motorium-related development, including gross motor development [22, 26, 31, 40], motor proficiency [29, 33, 37, 38], physical activity [20], ambulation [27] and muscle spasticity [32]. The second group of variables included body-related indicators, including cardiovascular function [24, 35], physical stress [25] and body function [28, 34, 39]. Lastly, a group of brain-related development variables could be categorised, including language development [36], brain activity [21, 34], salivary function [23] and neurodevelopmental progression [30, 36].

Regarding the methodological diversity of the papers, most studies (*N* = 15) can be considered randomised controlled trials [20, 21, 23, 25, 26, 30–37, 39], four of them were pilot (cross-sectional) studies [27–29, 40], one can be regarded as a non-randomised controlled trial [24] and one study did not report the type [22].

### Animals involved in the therapies

First, we checked the animals used in the programs. Two types of programs could have been detected based on the animal involved. Most articles included horse/equine-assisted activity/therapy/intervention (*n* = 16) [21–24, 27–32, 34, 35, 37–40]. The remaining five articles included canine-assisted activity/therapy/intervention [20, 25, 26, 33, 36].

Regarding the type of disorder, the papers had a different focus. Among the dog-assisted programs, autism [20], post-surgery status [25], cerebral palsy [26], developmental dysphasia [33] and severe and multiple disabilities [36] were represented. Among the equine-assisted programs, cerebral palsy gained significantly higher attention [21, 28, 31, 32, 35, 37, 40] and autism was also presented [39], and contrary to the dog-assisted programs, ADHD [29, 34], neurological disorders [24, 30], developmental delay [22], dyspraxia [27], physiological disorders [23], and physical disability [38] was also concerned. Therefore, we can see that equine-assisted programs were implemented in a broader context regarding the type of disorder.

### Age of the patients and the special needs represented

The age of the patients varied between 2 and 19 years. We could detect five groups of papers regarding the age of participants involved. Four papers involved *children participating in nursery education (2–6 years)*: Brady et al. [22] focused on children with developmental delay, Kraft et al. [30] on children with neurological disorders, Silkwood-Sherer & McGibbon [37] on those with cerebral palsy, and Machová et al. [33] on those with developmental dysphasia. Six papers focused on children belonging to the age bet*ween nursery and lower secondary education (2–14 years)*. Most of them involved children with cerebral palsy [21, 28, 31, 32], one on severe and multiple disabilities [36] and one on neurological disorders [24]. The biggest contrast in the age of the participants could be seen in the group of children belonging to the age between nursery and upper secondary education (2–18 years). In this group, two papers involved children diagnosed with cerebral palsy [26, 40], one with children in post-surgery status (unspecified disorders) [25] and one on those with physical disability [38]. One paper focused only on children belonging to th*e age of lower secondary education (10–13 years)*, known as the program of Steiner and Kertesz [39], focusing on participants with autism. The papers involving *children in primary and upper secondary education (6–19 years)* also showed a huge diversity. Two papers focused on children with attention deficit or/and hyperactivity disorder (ADHD) [29, 34], one on those diagnosed with cerebral palsy [35], one on those with autism [20], one on children with dyspraxia [27] and one those diagnosed with various physiological disorders [23].

Overall, we can state that studies focusing on lower secondary students are underrepresented. It is typical to involve patients belonging to diverse age groups into the studies although it would be important to note that huge differences can be hypothesised in the cognitive and social characteristics of children belonging to the different age groups.

### Session and program duration by age

**Fig. 2:**
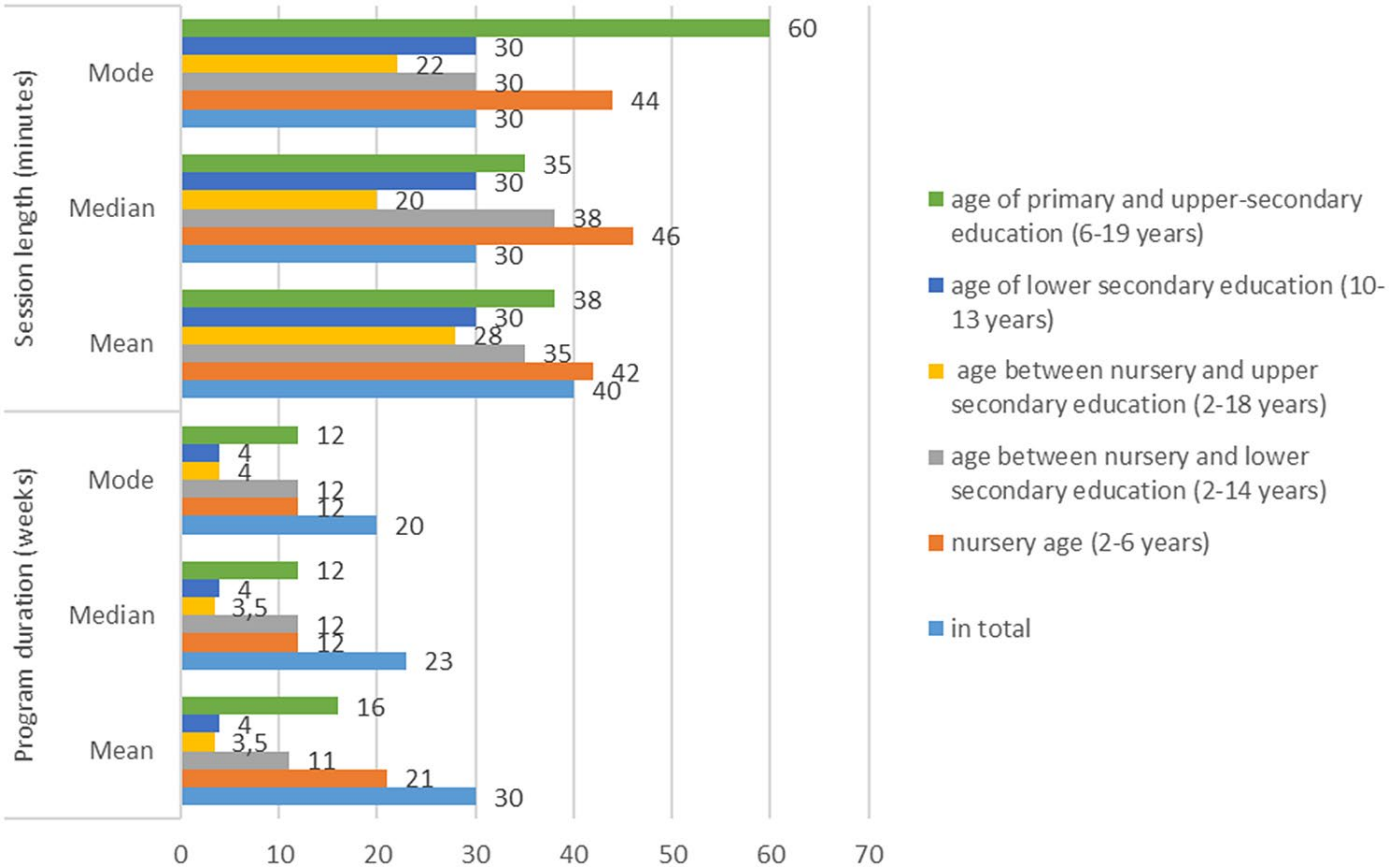
Means, median and modes of the programs by age

The range of the session length is between 8 and 60 min, the mean is 40 min, the median is 30 min, and the mode is 30 min as well. The range of the program duration is between 1 weeks and 4 years. The means, medians and modes of the programs divided by age are presented in Fig. 2. 

The session length of the programs focusing only on children in the *nursery age (2–6 years*) is usually long, three out of four last at least for 45 min [30, 33, 37] while having shorter, 15-30-minute-long sessions [22] is less typical. The length of the program is similar, it varies between 10 [33] and 12 weeks [30, 37] which is an average length overall. The range of the session length is between 25 and 50 min, the mean is 42 min, the median is 46 min, and the mode is 44 min as well. The range of the program duration is between 12 weeks and 40 weeks.

The session and program length showed a huge variety in the group of *children belonging to the age between nursery and lower secondary education (2–14 years).* Similarly to the programs created for nursery children, programs tend to be longer, 45 min [32, 36] rather than short [31] although the program of Benda et al. [21] is an exception with its 8-minute-long sessions. The average program duration was 12 weeks [28, 32, 36]. The range of the session length is between 8 and 50 min, the mean is 35 min, the median is 38 min, and the mode is 30 min as well. The range of the program duration is between 8 and 12 weeks.

In the group of *children belonging to the age between nursery and upper secondary education (2–18 years)*, a shift can be seen compared to the previously introduced lengths while shorter, 20-minute-long sessions [26] also appeared compared to the longer 45-minute-long sessions [38]. Also, shorter duration appeared [38], lasting for 6 weeks. The range of the session length is between 15 and 45 min, the mean is 28 min, the median is 20 min, and the mode is 22 min as well. The range of the program duration is between 1 weeks and 4 years.

The group of papers focusing on child*ren belonging to the age of lower secondary education (10–13 years)* showed incongruity while it contained only one program. This one [39] focused on 30-minute-long sessions through 4 weeks.

We could see shorter, medium-length and longer sessions and durations in the group of articles involving children in *primary and upper-secondary education (6–19 years)*. Despite the characteristics of the small school children, some programs reported having 60-minutes-long session [20, 34] which seems to be too long and not fitting the age group. Interestingly, significantly shorter, 10-minutes-long session could be found as well [23]. Overall, the duration varied between 8 and 12 weeks [27, 29] but longer formats also appeared [35]. The range of the session length is between 10 and 60 min, the mean is 38 min, the median is 35 min, and the mode is 60 min as well. The range of the program duration is between 7and 40 weeks.

### Outcome indicators

Regarding the outcome variables, two big categories could be created (see Table 2). *Physiological variables related to the nervous system* included papers measuring anthropometric variables, heart rate, respiratory rate, peripheral oxygen saturation (SpO2), systolic and diastolic blood pressure, brain activity, facial motricity, neurodevelopmental progression, physical stress and salivary cortisol. Among them, cerebral palsy [21, 35], developmental dysphasia [33], various physiological disabilities [23], post-operative cases [25], neurological disorder [24, 30] and severe and multiple disabilities were involved. Except for the trial of Cabiddu et al. [24], all papers were RCTs. Although the durations of the programs show a significant variety (between 1 occasion and 10 months), the length of the sessions was similar, approximately 45–50 min. Even if the papers focus on children with issues and the methods used also vary, the papers emphasise the positive impact of animal-assisted programs, supporting physiological health. The sympathetic nervous system works in a more efficient way, heart rate, respiratory rate, peripheral oxygen saturation (SpO2), systolic and diastolic blood pressure become slower as a result [24, 35]. Brain activity improves [10, 23] and neurodevelopmental progression can also be experienced [30, 36].

The other group of variables named as *motorium-related indicators* focused on children with autism [20, 39], developmental delay [22, 33], cerebral palsy [26, 28, 32, 35, 40], physical disability [38], ADHD [29, 34], dyspraxia [27]. Regarding the methodological diversity of these papers, RCTs were the most often used research method, except for pilot studies [27–29, 40]. The duration of the program showed a huge variety in this case as well since it varied between 2 weeks to 4 years. Regarding the session lengths, 30 and 45-minute-long sessions were preferred. Regarding the results, the papers highlight the positive impact of the animal-assisted activities, e.g. it improves motoric function and ambulation [28, 37, 38], gross-motor development and motor proficiency [22, 26, 31, 33], muscle spasticity [32] and coordination [34].

## Discussion

The results of the systematic literature analysis highlighted the common health outcomes addressed by animal-assisted interventions. Regarding the effects on the nervous and motoric systems, an overall positive and supportive impact could be detected compared to the control groups receiving normal pharmacological therapy. The significance of the impact may vary following the type of the disorder, its nature, severity and comorbidity. The papers focused rather on physical disabilities (e.g. cerebral palsy, dysphasia, etc.), and the manifestation of developmental disorders (autism, ADHD) was lower, probably due to the physiological improvement focus of the articles. Multidisciplinary models support the complex treatment of physiological and psychological aspects of disorders and active participation in the medical process by child psychiatrists and general paediatricians, some of whom act as intermediaries between the health care, public education and social sector [41]. For instance, according to Nagy et al. [42], in addition to parents and specialists, the involvement of child psychiatrists and general paediatricians may be justified, as they act as intermediaries between the health care, public education and social sectors. Therefore, future research should emphasise the correlation between various physiological and psychological parameters in the light of the therapy as understanding these interconnected aspects can provide a more comprehensive insight into the mechanisms and effectiveness of therapeutic interventions. Physiological responses, such as changes in heart rate, cortisol levels, and immune system function, are intricately linked with psychological well-being. Investigating the correlations between these physiological markers and psychological outcomes can unveil the intricate interplay between mind and body during therapeutic processes. This holistic approach is particularly relevant in the field of therapy, where interventions often aim to address both the mental and physical aspects of health [43].

During the analysis, the consensus could be drawn regarding the length of the sessions that the length of the sessions is below 60 min. Partly, the variety of the disorders manifested in the programs hinders concluding such suggestions. However, no consensus could be seen in the case of similar disorders. Also, a minimum of 6 weeks would be important when operating with one or two sessions per week. This would also improve the development of the therapeutic bond, deepening the impact of the activity. However, the study of Odendaal [44] reported the more beneficial impact of the more frequent shorter dog-assisted program (5–25 min) compared to less frequent longer ones (60 min). Therefore, sessions should follow this suggestion and set the main animal-assisted task into shorter periods. Also, it would be important to set the duration and length following the age and disorder of the patients.

**As implications for practice**, this systematic analysis provides beneficial information for clinicians, healthcare providers, and education providers, supporting the identification of various physiological impacts of animal-assisted programs (interventions, therapies, activities). This incorporates developing or ensuring the application of the needs assessment checklists, sampling, timing including session length and duration, choice of animals and measurement tools. The conclusions drawn regarding the length and durations of the various programs may support professionals to design their therapeutic activities. The programs introduced may also support professionals in the choice of the most appropriate animal-assisted program following the special need and health problem and the age of the patients.

Despite the findings of this study, it is important to recognise the limitations that it presents. For instance, there were variances between trials that could not be fully clarified by the moderator analyses of the trial characteristics since the studies used different tools or focused on different patient groups. While we did gather extensive data on factors such as the quality of the trial, the participants, and the interventions, there remained some unaccounted-for heterogeneity in the trials. Due to the heterogeneity of the studies, no pool sizes and effect sizes could be measured. Also, since the language of the papers was only English, some valuable articles written in non-English language were not considered. Also, some relevant articles were not included due to the search process used. Also, grey literature was excluded. We also emphasise that these results only represent published studies and they do not represent unpublished research or what may be done in actual practice.

We should also mention that several studies have utilised interventions incorporating multiple treatment methods. This has led to the difficulty of categorising these studies into distinct groups. Additionally, some studies have not furnished sufficient information regarding their treatment approaches and delivery methods. Treatment protocols must be more transparently documented in the primary research literature to explore the most effective elements of psychotherapies for mitigating symptoms and enhancing everyday activities in children and youth with special needs. Also, we should mention the diversity of the keywords that could have been potentially used during the systematic search, e.g. “behavioural intervention”, “treatment” or “strategy” instead of “intervention”.

## Conclusions

This study identified several issues related to animal—assisted interventions, therapies and programs focusing on the physiological development of children and youth with special needs. These were comparable to studies in other settings and countries. The methodology of the papers represents a high niveau. There is still a gap in the development of such programs since existing activities, therapies, and interventions focus only on the nervous system and the moratorium. The interrelation of the physiological and psychological variables is not dominant at all. Identifying these problems can provide useful information to inform recommendations for strategies to enhance the practice and management of animal-assisted programs focusing on children and youth with special education needs. Enhancing the practice and management of animal-assisted programs involves careful consideration of various strategies to ensure the well-being of both animals and participants. Based on the findings, standardised training programs can be established for handlers and animals involved in animal-assisted programs. Certification processes can ensure that handlers are knowledgeable about animal behaviour, welfare, and the specific needs of diverse populations. Regarding methodological aspects, the relevance of the screening progress may also be emphasised as implement thorough screening processes for participants to identify any allergies, fears, or health conditions that may affect their interaction with animals. Therefore, further research should also put bigger focus on such details. This helps tailor the program to individual needs and ensures a positive experience. Also, ongoing evaluation processes are also crucial to assess the effectiveness and impact of animal-assisted programs. Solicit feedback from participants, handlers, and other stakeholders to continuously improve program quality. Furthermore, fostering collaboration between animal-assisted programs and relevant professionals, such as psychologists, veterinarians, and educators should also have higher emphasis. This interdisciplinary approach ensures a comprehensive understanding of the benefits and challenges associated with these programs.

### Electronic supplementary material

Below is the link to the electronic supplementary material.

Supplementary Material 1

## Data Availability

The datasets used and/or analysed during the current study available from the corresponding author on reasonable request.
