# Supplementary material for: The role of animal-assisted programs in physical health improvement of children and adolescents with special education needs - a systematic review
Source: BMC Public Health. 2024 Mar 15;24:824. doi: 10.1186/s12889-024-18326-y (PMC10943833; doi:10.1186/s12889-024-18326-y)
Supplement: Supplementary file 1 — Supplementary Material 1 [file 12889_2024_18326_MOESM1_ESM.docx]

**Appendix**

**Supplementary Table 1.** The risk assessment of randomized controlled trials

|  | **Q1** | **Q2** | **Q3** | **Q4** | **Q5** | **Q6** | **Q7** | **Q8** | **Q9** | **Q10** | **Q11** | **Q12** | **Q13** | **Risk assessment** |
| --- | --- | --- | --- | --- | --- | --- | --- | --- | --- | --- | --- | --- | --- | --- |
| Benda et al. [10] | Yes | Yes | Yes | Yes | Yes | Yes | Yes | Yes | Yes | Yes | Yes | Yes | Yes | Low |
| Brady et al. [11] | Yes | Yes | Unclear | Unclear | Unclear | Yes | Yes | Yes | Yes | Yes | Yes | Yes | Unclear | Moderate |
| Branson et al. [12] | No | No | Yes | Unclear | Unclear | No | Yes | Yes | Yes | Yes | Yes | Yes | Yes | Moderate |
| Cabiddu et al. [13] | Yes | Unclear | Yes | No | No | Unclear | Yes | Yes | Yes | Yes | Yes | Yes | Yes | Moderate |
| Calcaterra et al. [14] | Yes | Yes | Yes | Unclear | Unclear | Yes | Yes | Yes | Yes | Yes | Yes | Yes | Yes | Moderate |
| Deutz et al. [15] | Yes | Yes | Yes | No | No | Yes | Yes | Yes | Yes | Yes | Yes | Yes | Yes | Low |
| Kraft et al. [19] | Yes | Yes | Yes | Yes | Yes | Yes | Yes | Yes | Yes | Yes | Yes | Yes | Yes | Moderate |
| Kwon et al. [20] | Yes | Yes | Yes | Yes | Yes | Yes | Yes | Yes | Yes | Yes | Yes | Yes | Yes | Low |
| Lucena-Antón et al. [21] | Yes | Yes | Yes | Yes | Yes | Yes | Yes | Yes | Yes | Yes | Yes | Yes | Yes | Low |
| Machová et al. [22] | Yes | No | Yes | Unclear | Unclear | Yes | Yes | Yes | Yes | Yes | Yes | Yes | Yes | Low |
| Oh et al. [23] | No | Unclear | Yes | Yes | Yes | Unclear | Yes | Yes | Yes | Yes | Yes | Yes | Yes | Low |
| Park et al. [24] | Yes | Unclear | Yes | Unclear | Unclear | Yes | Yes | Yes | Yes | Yes | Yes | Yes | Yes | Low |
| Rincón et al. [25] | Yes | Yes | Yes | Unclear | Unclear | Yes | Yes | Yes | Yes | Yes | Yes | Yes | Yes | Low |
| Silkwood-Sherer & McGibbon 26 | Yes | Unclear | Yes | Unclear | Unclear | Unclear | Yes | Yes | Yes | Yes | Yes | Yes | Yes | Moderate |
| Silkwood-Sherer et al. [27] | Yes | Unclear | Yes | Unclear | Unclear | Unclear | Yes | Yes | Yes | Yes | Yes | Yes | Yes | Moderate |
| Steiner & Kertesz [28] | Yes | Yes | Yes | Yes | Yes | Yes | Yes | Yes | Yes | Yes | Yes | Yes | Yes | Low |

Q1. Was true randomisation used for assignment of participants to treatment groups? Q2. Was allocation to treatment groups concealed? Q3. Were treatment groups similar at the baseline? Q4. Were participants blind to treatment assignment? Q5. Were those delivering treatment blind to treatment assignment? Q6. Were outcomes assessors blind to treatment assignment? Q7. Were treatment groups treated identically other than the intervention of interest? Q8. Was follow-up complete, and if not, were differences between groups in terms of their follow-up adequately described and analysed? Q9. Were participants analysed in the groups to which they were randomised? Q10. Were outcomes measured in the same way for treatment groups? Q11. Were outcomes measured in a reliable way? Q12. Was appropriate statistical analysis used? Q13. Was the trial design appropriate, and any deviations from the standard RCT design (individual randomisation, parallel groups) accounted for in the conduct and analysis of the trial?

**Supplementary Table 2.** The risk assessment of pilot studies

|  | **Q1** | **Q2** | **Q3** | **Q4** | **Q5** | **Q6** | **Q7** | **Q8** | **Risk assessment** |
| --- | --- | --- | --- | --- | --- | --- | --- | --- | --- |
| Hession et al. (2014) | Yes | Yes | Yes | Yes | Yes | Yes | Yes | Yes | Low |
| Hsieh et al. (2015) | Yes | Yes | Yes | Yes | No | No | Yes | Yes | Low |
| Jang et al. (2015) | Yes | Yes | Yes | Yes | No | No | Yes | Yes | Low |
| Žalienė et al. (2018) | Yes | Yes | Yes | Yes | Yes | Yes | Yes | Yes | Low |

Note: Questions of JBI are as follows: Q1. Were the criteria for inclusion in the sample clearly defined? Q2. Were the study subjects and the setting described in detail? Q3. Was the exposure measured in a valid and reliable way? Q4. Were objective, standard criteria used for measurement of the condition? Q5. Were confounding factors identified? Q6. Were strategies to deal with confounding factors stated? Q7. Were the outcomes measured in a valid and reliable way? Q8. Was appropriate statistical analysis used?
